# Care of Adults with Advanced Chronic Kidney Disease

**DOI:** 10.3390/jcm13154378

**Published:** 2024-07-26

**Authors:** Sanjivani Shrestha, Kanza Haq, Divyanshu Malhotra, Dipal M. Patel

**Affiliations:** Division of Nephrology, Johns Hopkins University School of Medicine, Baltimore, MD 21287, USAdmalhor2@jh.edu (D.M.)

**Keywords:** non-dialysis-dependent CKD, pre-dialysis care, supportive management

## Abstract

Chronic kidney disease (CKD) impacts over 10% of the global population. Adults with CKD face significant morbidity and mortality. As kidney disease progresses, the risk of adverse outcomes increases. Here, we present an overview of strategies to care for adults with advanced CKD (stage 4–5 CKD, not receiving kidney replacement therapy). We aim to guide clinicians through several aspects of CKD care, ranging from recommended laboratory assessments to interdisciplinary support for patients as they plan for kidney replacement therapy (dialysis, transplantation, or conservative management). We incorporate considerations of health equity and person-centered care, empowering clinicians to deliver high-quality care to people with CKD.

## 1. Introduction 

Chronic kidney disease (CKD) is a highly prevalent condition, impacting over 10% of the worldwide population, and is anticipated to be the fifth leading cause of mortality by 2040 [1]. Management of common risk factors for kidney disease including hypertension and diabetes, and avoidance of nephrotoxic agents, can sometimes stabilize kidney function. However, in many cases CKD is a progressive condition associated with outcomes of kidney failure, cardiovascular events, and mortality. The incidence of these outcomes is highest for people with advanced CKD, defined here as people with a glomerular filtration rate (GFR) < 30 mL/min/1.73 m^2^ who have not initiated kidney replacement therapy. Care of people with advanced CKD involves the management of risk factors as well as attention to anemia, acid-base balance, mineral bone disease, and impairments to quality of life that commonly arise in this population.

**Figure 1:**
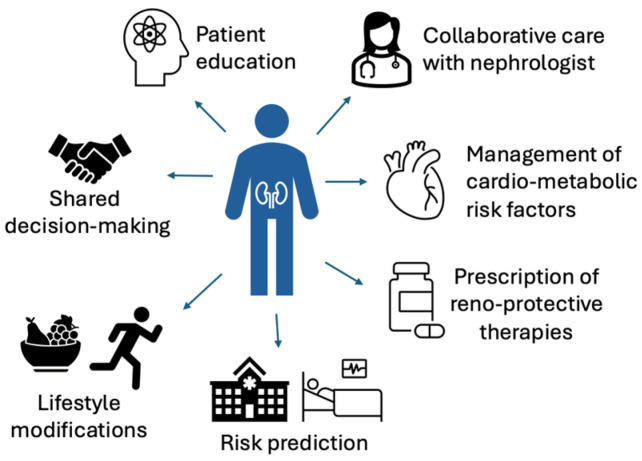
Multifaceted care of adults with advanced CKD.

In this article, we provide an overview of strategies to monitor kidney function, manage associated impairments, and provide support as kidney function may worsen for people with advanced CKD (Figure 1). Collaborative care between clinicians across multiple specialties of medicine is needed to appropriately manage this growing population.

## 2. Epidemiology of CKD

### 2.1. Prevalence of CKD

Worldwide, approximately 850 million people are estimated to have kidney disease [2]. The global prevalence of CKD is roughly double that of diabetes and 20 times more than the prevalence of cancer [3]. The prevalence of CKD is growing due to concomitant increases in common risk factors such as diabetes and obesity [1]. A systematic review and meta-analysis of >6.9 million people conducted in 2016 calculated the prevalence of CKD G3 as 7.6% of the worldwide population, whereas advanced CKD was less common (0.5% for CKD G4–5) [4]. 

### 2.2. Risk Factors for CKD Development and Progression

The common risk factors for the development of CKD include diabetes, high blood pressure, obesity, and cardiovascular disease [3]. The increase in the prevalence of these risk factors likely contributes to the growing prevalence of CKD. Additional risk factors include environmental exposures, dehydration, infection, use of nephrotoxic agents, and genetic conditions, many of which will not be adequately recognized unless people are screened for CKD with laboratory measurements, including urinalysis [2].

CKD prevalence is estimated to be higher in older people and in females [1]. However, the definition of CKD in older adults [5], and the balance between a higher prevalence of CKD in women with the known protective benefits of estrogen, which may contribute to slower disease progression in females [6], are controversial. CKD prevalence and severity can also vary significantly based on race, ethnicity, and social determinants of health. In general, CKD disproportionately affects poor and marginalized populations [2]. In the U.S., the incidence of CKD is higher in Hispanic individuals and in individuals with lower income and education compared to individuals with higher income and education [3]. Furthermore, a disproportionate number of Black people with CKD experience a progression of disease to kidney failure [3]. 

### 2.3. Access to Care

The prevalence of CKD is higher in low- and middle-income countries. However, the availability of nephrology care and services including kidney replacement therapy (KRT) is variable. In a robust literature review of data from 161 countries, 98% of countries offered hemodialysis, whereas 79% offered peritoneal dialysis and 70% offered kidney transplantation [7]. In many countries, access to KRT is dependent on out-of-pocket and private payments [8]. The care of people with advanced CKD must be tailored to resources available in the local care environment. Furthermore, attention is needed to increase the awareness of CKD, as many people with CKD are unaware of their diagnosis and do not seek treatment [2]. 

### 2.4. Global Impact of CKD

People with CKD are at high risk for morbidity and mortality, and CKD is predicted to be the fifth highest cause of mortality by 2040 [9]. Healthcare costs for people with CKD exceed > $85.4 billion in the U.S. alone, with most costs going towards people with kidney failure [10]. Reducing the burden of CKD will require multidimensional and interdisciplinary care to address common and uncommon risk factors for kidney disease, as well as the social drivers of inequity and access to care [9]. 

## 3. CKD Staging and Prognosis 

### 3.1. Diagnosing CKD

CKD is diagnosed by a decline in kidney function for ≥3 months, or by the presence of structural/pathologic abnormalities of kidney tissue as identified by kidney biopsy, imaging studies, or urinary studies (Table 1) [3]. In the absence of other markers of kidney damage, a GFR above 60 mL/min does not fulfill the diagnostic criteria for CKD. Additional guidelines on detection and evaluation of CKD can be found elsewhere [3,11]. 

### 3.2. Assessment of the Estimated GFR (eGFR)

Serum creatinine (Cr) is the endogenous serum filtration marker most commonly used to calculate eGFR_cr_ using one of several estimating equations [3]. Cr has several potential limitations, including its dependence on non-GFR determinants such as body habitus, muscle mass, eating habits, and medications that alter the tubular secretion of Cr. Serum cystatin C (Cys) has recently gained recognition as an alternative filtration marker that can improve the performance of estimating equations [12]. It is recommended to calculate eGFR_cr-cys_ when eGFR_cr_ is felt to be less accurate or when GFR estimates can impact clinical decision-making [3]. 

Utility of mGFR: Both Cr and CysC are serum filtration markers, which can be less accurate in settings of catabolic states, inflammation, use of high-dose steroids, frailty, and high cell turnover. Therefore, in conditions such as malnutrition, cancer, heart failure, cirrhosis, and muscle wasting diseases, the calculations of measured GFR (mGFR) using plasma or urinary clearance of an exogenous filtration marker such as iohexol can be pursued to maximize accuracy [3].

Race-free estimates: The Cockcroft–Gault, Modification of Diet in Renal Disease (MDRD) Study, and the 2009 Chronic Kidney Disease Epidemiology Collaboration (CKD-EPI) eGFR equations included race as a coefficient to calculate eGFR for any given Cr value. As a result, people who are Black or African American were estimated to have a higher eGFR than other individuals, which contributed to disparities in health and health care delivery in Black and African American communities [13]. Black and African American patients historically received late referrals for nephrology care, which has been associated with a decrease in survival [14]. The 2021 CKD-EPI eGFR_cr_ and eGFR_cr-cys_ provide race-free estimates, with an improvement in accuracy and less difference in eGFR calculations between people with CKD of different races. It is predicted to result in new CKD diagnoses for >400,000 Black adults in the U.S. and to reclassify >500,000 Black adults to advanced stages of CKD [15], both of which can help ensure that these patients can appropriately be referred for nephrology care and KRT planning (including transplantation).

Considerations for older adults: eGFR may slowly decrease with aging as a result of expected cellular and organ senescence [5]. The standard interpretation of eGFR values in this population may result in the overdiagnosis of CKD [5]. These findings have led to discussions of age-calibrated definitions of CKD in which the GFR threshold to define CKD is lowered to 45 mL/min for adults >65 years old [16]. Eliminating a diagnosis of CKD for older adults could reduce unjustified stress and difficulties surrounding healthcare and life insurance [17]. Notably, epidemiologic studies have demonstrated that older adults with an eGFR between 45–60 mL/min/1.73 m^2^ are at a higher absolute risk of death than of kidney failure [3,18]. Therefore, while this population deserves thoughtful attention to risk mitigation strategies, clinical care may be less focused on reducing kidney outcomes. 

### 3.3. Assessment of Albuminuria

Albuminuria is associated with a graded increase in risk for mortality, progression of CKD, and kidney failure, independent of eGFR [19]. The early diagnosis of albuminuria via UACR measurement can result in the early initiation of proteinuria-reducing therapies such as renin-angiotensin system inhibitors (RASi), mineralocorticoid receptor antagonists (MRA), and sodium-glucose cotransporter-2 inhibitors (SGLT2i). While albuminuria is the preferred measurement for definition and staging of CKD, total urine protein or dipstick protein are often measured and can be converted to UACR using an estimating equation [20]. 

### 3.4. The CKD G/A Classification System

**Figure 2:**
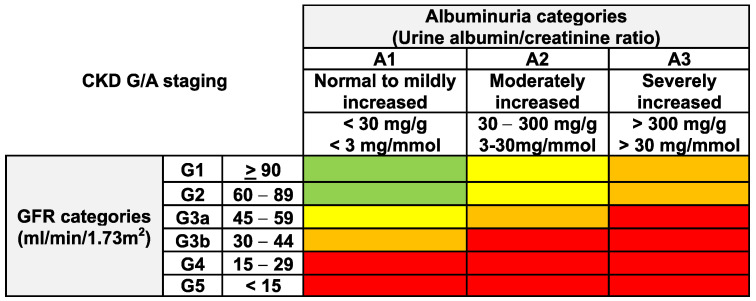
CKD heatmap, demonstrating the increase in risk of adverse outcomes for people with lower GFR and higher albuminuria levels [3]. Risk of adverse outcomes is low (green), moderately increased (yellow), high (orange), or very high (red). GFR: glomerular filtration rate.

Given the clear association of adverse outcomes with kidney function as well as albuminuria levels, international guidelines advocate for use of the “CGA” classification system, which includes CKD etiology (C), GFR category (G), and albuminuria category (A) [3] (Figure 2). The etiology of CKD can be assessed by obtaining a relevant history (e.g., review of systems, comorbidities, surgical history, family history, medication and supplement use), laboratory tests including serologic tests, examination of urine sediment, imaging, and, in some cases, genetic testing or kidney biopsy [3,11]. 

### 3.5. Risk Prediction

The prognosis of people with CKD for adverse outcomes including all-cause mortality, hospitalizations, cardiovascular events, and progression to kidney failure is associated with CKD staging, as commonly displayed on CKD “heatmaps” [3,18] (Figure 2). As reviewed later in this article, heatmaps can guide clinicians in determining a recommend frequency of laboratory checks. 

Since there can be significant variability in the risk of CKD progression or kidney failure for two people in the same heatmap category, individual risk prediction using validated risk equations can also be helpful in counseling and caring for individuals with CKD [21]. Validated risk equations include the Kidney Failure Risk Equation (KFRE), which predicts an individual’s risk of progressing to kidney failure within the next 2 or 5 years [22]. The four-variable KFRE calculates risk based on age, sex, estimated GFR (eGFR), and urine albumin-creatinine ratio (UACR), whereas the eight-variable equation further incorporates serum albumin, phosphate, calcium, and bicarbonate levels. Other relevant risk prediction models include the Advanced CKD Risk Tool [23], which predicts the 2-year and 4-year risk of kidney failure as well as the risk of cardiovascular disease and death, and a model that predicts the 3-year risk of ≥40% decline in kidney function [24]. The predicted risk should be recalculated over time, especially with significant changes in patients’ health or after acute events such as hospitalizations.

### 3.6. Defining Advanced CKD

People with advanced CKD are at the highest risk of adverse events, including progression to kidney failure, hospitalization, and mortality. Advanced CKD typically includes people with an eGFR < 30 mL/min/1.73 m^2^ [25]. Given a competing risk of death in this population, it is suggested that the eight-variable 2-year KFRE be used to predict the risk of kidney failure over the short-term, whereas the 4-year Advanced CKD Risk Tool (which accounts for the competing risk of death) can be used to predict the risk of kidney failure over a longer term [26]. In addition to preparing people with advanced CKD for KRT, treatment goals center around the management of disease complications such as hypertension, anemia, mineral bone disease, and acidosis. The details of these strategies are outlined later in this article. 

## 4. Management of People with Advanced CKD

### 4.1. Suggested Frequency of Lab Assessment and Clinic Visits

A suggested frequency of lab measurements for people who are diagnosed with CKD is shown in Table 2 [3]. Renal function panels should include measurements of eGFR_cr_ or eGFR_cr-cys_, as noted above, as well as measurements of serum phosphorus and calcium levels. In settings of anemia and/or significant fatigue, iron panels can be checked along with complete blood counts. Depending on provider preference, Vitamin D levels can be checked to assess for reversible causes of secondary hyperparathyroidism, as discussed below. 

Laboratory assessment should also be more frequent in select circumstances: (1) when an individual is noted to have a > 20% decline in eGFR; (2) when an individual is noted to have a doubling of UACR; and (3) when an individual experiences a > 30% reduction in eGFR after the initiation of hemodynamically active therapies including RASi, MRA, and SGLT2i [3]. If a patient is being treated with erythropoiesis-stimulating agents for anemia, hemoglobin is typically checked monthly. 

The frequency of clinic visits may align with the frequency of laboratory assessment, though people with progressive CKD G5 often require more frequent (monthly) clinic visits to adequately prepare for transitions to KRT. The frequency of monitoring may also depend on resource availability, patient preference, and cost. 

### 4.2. Lifestyle Modifications

People with advanced CKD should be encouraged to maintain healthy lifestyles, which include (1) physical activity with at least 150 min per week of moderate intensity exercise; (2) achievement of optimal body mass index; (3) avoidance of tobacco products; and (4) maintenance of a healthy diet that limits sodium intake (<2 g per day), prioritizes plant-based foods, and limits ultra-processed foods [3]. 

There is increasing evidence that plant-based diets may offer multiple benefits of slowing the progression of CKD, decreasing the incidence cardiovascular disease, reducing the rates of diabetes and obesity, and reducing inflammation and cholesterol, which in turn can delay the onset of kidney failure and the initiation of dialysis [27,28]. While the ingestion of animal-based proteins can promote an acidic environment, inflammation, and renal hyperfiltration, plant-based proteins can be alkaline-forming, anti-inflammatory, and reno-protective [29]. 

Guidance on protein intake is somewhat controversial, but, in most cases, people with advanced CKD are recommended to maintain a protein intake under 0.8–1.0 g/kg body weight [30]. In select people with advanced CKD who are metabolically stable and can be closely monitored, very low-protein diets of 0.3–0.4 g/kg body weight can be considered [31]. Plant-based proteins are favored, as above. There are several methods that can be used to assess each individual’s dietary intake, ranging from traditional methods such as food diaries to novel methods such as image-assisted dietary assessment, wearable technologies, and the assessment of the biomarkers of dietary intake (e.g., 24 h urinary nitrogen, which can reflect protein intake). The assessment of dietary intake can help form more personalized dietary recommendations for each individual with CKD.

### 4.3. Guideline-Directed Medical Therapies to Reduce Proteinuria

Proteinuria is a strong risk factor for CKD progression, cardiovascular (CV) disease, and death [32,33,34]. The Kidney Disease Improving Global Outcomes (KDIGO) guidelines advocate for the use of proteinuria-reducing therapies for people with kidney disease of multiple etiologies ranging from diabetic kidney disease to glomerular disease [3,35,36]. Proteinuria-reducing therapies lower the risk of reaching kidney failure, in addition to conferring benefits to blood pressure control, glycemic control, and CV health in a population commonly impacted by multiple comorbidities. Thus, proteinuria-reducing therapies are a mainstay of guideline-directed medical therapy for people with kidney disease, heart disease, and diabetes (Table 3).

Historically, the therapeutic options to reduce proteinuria were limited to RASi or MRA, both of which can result in hyperkalemia, especially for people with advanced kidney disease and/or diabetes. The discontinuation of these therapies, which is a common strategy for management of their associated hyperkalemia [39], results in a higher risk of cardiorenal events, including heart failure hospitalizations and the progression of kidney disease [40,41]. The availability of newer potassium-binding resins may enable the continued use of RASi and MRA for patients prone to hyperkalemia [42]. The potential benefits of such strategies are significant, as continuation of RASi (even in CKD G4-5) has been shown to reduce mortality and major adverse CV events (MACE) [43]. Newer non-steroidal MRAs are shown to lower rates of kidney disease progression and CV events [44], with a lower side effect profile than steroidal MRAs. The data on kidney outcomes for people with advanced CKD have been mixed, with some studies indicating that discontinuing RASi for people with advanced CKD results in a reduction of the risk of progressing to kidney failure [43], and others demonstrating that continuation of RASi slightly decreases the risk of reaching kidney failure [45]. The potential risks of RASi and MRA may contribute to provider hesitancy to prescribe these therapies for people with CKD. However, since CV disease is the leading cause of death for people with CKD [46], the continuation of RASi and MRA is warranted for their CV and mortality benefit. 

Sodium-glucose cotransporter 2 inhibitors (SGLT2i) and glucagon-like peptide 1 receptor agonists (GLP-1 RA) are newer agents that were initially developed for the treatment of diabetes. Subsequent studies have demonstrated the clear benefit of these agents in reducing proteinuria and protecting kidney function. SGLT2i and GLP-1 RA are both included in subspecialty guidelines as evidence-based therapies for the management of people with diabetic kidney disease [36,47], and ongoing studies are evaluating their impact in non-diabetic and non-proteinuric kidney disease. The CV benefits of these agents are also abundant. SGTL2i are included as guideline-directed medical therapy for people with heart failure as a means to reduce CV mortality and hospitalization risk [37]. GLP-1 RA reduce MACE and heart failure hospitalizations [48,49]. Semaglutide was recently shown to reduce progressive decline in kidney function, as well as reducing cardiovascular mortality, in people with diabetes and CKD [50]. SGLT2i can be initiated for people with an estimated glomerular filtration rate (eGFR) ≥ 20 mL/min/1.73 m^2^ (and continued if eGFR drops < 20 mL/min/1.73 m^2^), whereas GLP-1 RA use is not restricted by kidney function. Ongoing trials studying SGLT2i and GLP-1 RA are anticipated to expand upon the benefit of these agents for kidney and CV outcomes.

The implementation of guideline-adherent care is of key importance. The challenges in implementation include low rates of screening for comorbidities and kidney disease, the lack of provider comfort with guidelines, high rates of drug discontinuation, and cost [51]. Involvement of primary care physicians and pharmacists in following guidelines has been shown to be successful [52,53]. 

### 4.4. Management of Hypertension 

In general, adults with CKD are recommended to maintain systolic blood pressure (BP) < 120 mmHg (or < 130 mmHg for people with kidney transplants) using standardized office BP measurements [54,55]. This recommendation is balanced by a need for less intensive BP targets for adults who are frail, at high risk for falls or fractures, have limited life expectancy, or have symptomatic postural hypotension [56]. The guidelines focus on systolic targets given wide pulse pressure variations in adults with CKD, with an assumption that if systolic BP is <120 mmHg, diastolic BP will typically be <70 mmHg. 

We note some discrepancy between the Kidney Disease Improving Global Outcomes (KDIGO) and American College of Cardiology/American Heart Association (ACC/AHA) guidelines, with ACC/AHA guidelines suggesting a BP target of <130/80 mmHg for people with CKD [55,56]. We also note that in the post-hoc analysis of the Systolic Blood Pressure Intervention Trial (SPRINT), intensive anti-hypertensive treatment targeting a systolic BP < 120 mmHg was associated with a higher risk of eGFR decline and of kidney failure, though these adverse outcomes were not seen after the intervention phase [57]. This potential risk of intensive BP lowering is especially important to consider in adults with advanced CKD. 

KDIGO guidelines advocate for use of RASi for people with CKD and diabetes and for people with CKD and albuminuria [55], though there are some potential limitations surrounding kidney outcomes with RASi initiation in people with advanced CKD (see section on proteinuria-reducing therapies). Other commonly prescribed anti-hypertensive agents include thiazide-type diuretics (which are effective for BP control in advanced CKD [58]) and calcium channel blockers, as well as beta-blockers and loop diuretics, depending on each individual’s comorbidities and needs. We refer to detailed guidelines and evidence on the use of each agent, summarized elsewhere [55,56].

### 4.5. Management of Anemia

People with advanced CKD commonly experience anemia, in part due to the loss of endogenous erythropoietin production from kidneys [59]. Iron deficiency is also common due to frequent blood sampling for laboratory testing, blood loss from surgical procedures (such as the creation of vascular access), interference with iron absorption due to medications such has gastric acid inhibitors and phosphate binders, and limited iron absorption due to inflammation [60]. Anemia is a condition that can be treated to improve the quality of life for people with CKD. In fact, treatment of iron deficiency, even in the absence of anemia, has been shown to result in an improvement in energy levels for people with CKD [61,62]. Though treatment with iron and erythropoiesis-stimulating agents (ESAs) have some associated costs and risks, they are generally favored as a means to avoid blood transfusions, especially in transplant candidates, given the risk of antibody formation from blood transfusions, which can limit compatibility with potential kidney allografts. 

**Figure 3:**
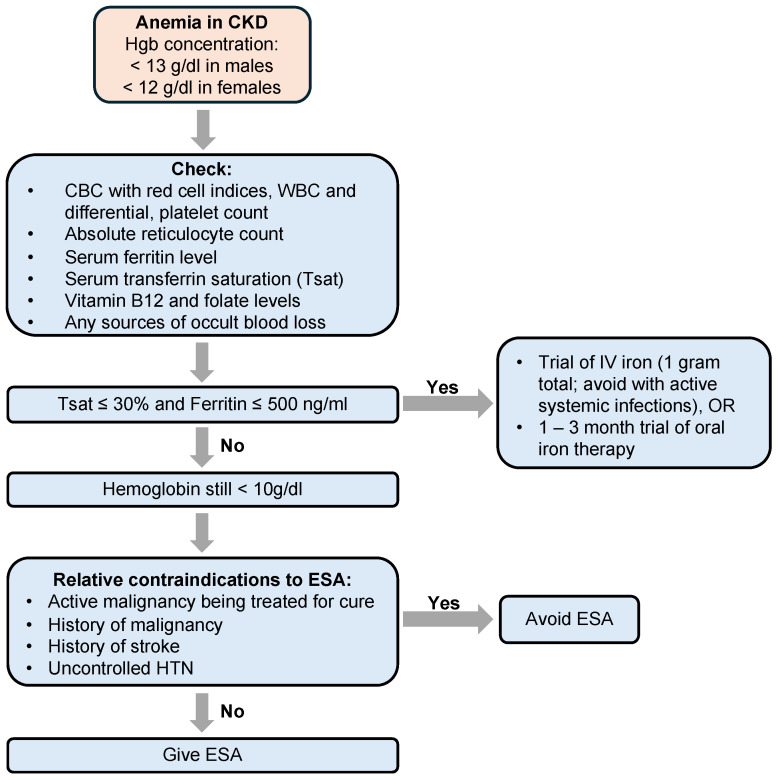
Algorithm for evaluation and management of anemia in CKD. ESA: erythropoiesis-stimulating agent.

When people with CKD are found to have anemia, guidelines recommend testing for specific causes of the anemia, which include iron, vitamin B12, and folate deficiency, as well as sources of occult blood loss [63] (Figure 3). If iron deficiency is identified (transferrin saturation level ≤30% and a ferritin level ≤500 ng/mL), iron repletion is recommended and can sometimes require the use of IV iron infusions [64]. Erythropoiesis-stimulating agents can be initiated when iron stores are replete but hemoglobin is still <10 g/dL. The use of ESAs is relatively contraindicated for people with an active malignancy being treated with a curative intent, a history of malignancy, a history of stroke, or uncontrolled HTN [65]. The risks and benefits of using oral hypoxia-inducible factor-prolyl hydroxylase enzyme inhibitors (HIF-PHI) for people with non-dialysis-dependent CKD are being actively investigated [66,67].

### 4.6. Management of Mineral Bone Disease

People with advanced CKD are at high risk for CKD mineral bone disease (CKD-MBD), which is often associated with secondary hyperparathyroidism (SHPT) [68]. Mechanistically, the development of CKD-MBD involves early increases in the levels of fibroblast growth factor 23 (FGF23), which is released from osteocytes [69,70]. FGF23 promotes renal phosphate excretion, which may explain the maintenance of normal serum phosphate levels even as eGFR declines. FGF23 also reduces levels of 1.25 (OH)2 vitamin D (calcitriol), which results in a reduction in serum calcium levels. The ensuing hypocalcemia stimulates the increased release of intact parathyroid hormone (PTHi). Hyperphosphatemia is typically seen in the later stages of this pathway, likely when the phosphaturic signals of both FGF23 and PTHi are limited by diminishing renal capacity to excrete phosphorus. In total, laboratory parameters seen in people with CKD-MBD can include low calcitriol, hypocalcemia, elevated PTHi, and hyperphosphatemia.

CKD-MBD and uncontrolled SHPT can be associated with an increased rate of fractures, cardiovascular events, and mortality [68]. Even in non-dialysis-dependent CKD, CKD-MBD can manifest as calcific uremic arteriolopathy (CUA, also referred to as calciphylaxis), in which calcium accumulates throughout small blood vessels, resulting in exquisitely painful lesions that a carry high risk of infection. Management of CKD-MBD is therefore a key component of care for people with advanced CKD. However, the enthusiasm to treat biochemical parameters of CKD-MBD is dampened by a lack of strong evidence that treatment actually reduces mortality or improves person-centered outcomes. 

Still, KDIGO guidelines recommend that in people with non-dialysis-dependent CKD, (1) hypercalcemia should be avoided given its association with vascular calcifications and cardiovascular disease; (2) hyperphosphatemia should be avoided through dietary modifications and (if needed) phosphate-lowering therapies taken with food; and (3) though the optimal PTHi level is not known, modifiable risk factors (hypocalcemia, hyperphosphatemia, high phosphorus intake, or Vitamin D deficiency) can be treated if PTHi levels are progressively rising or persistently elevated [68]. Furthermore, bone mineral density testing should be pursued to assess for fracture risk, especially when the results of testing will influence treatment decisions. 

### 4.7. Management of Acidosis 

CKD can be associated with the impaired excretion of hydrogen ions. The resultant metabolic acidosis causes a range of adverse health outcomes including worsening cardiovascular status, impaired bone mineral density, and the progression of kidney disease [71]. Bicarbonate supplementation, often given in the form of sodium bicarbonate tablets, consumption of baking soda, or through dietary modifications including increased intake of citrus (e.g., lemon water), can be used to mitigate metabolic acidosis. 

However, in a placebo-controlled trial of oral sodium bicarbonate conducted by the BiCARB study group, which studied people with CKD G3-5 aged ≥ 60 years and serum bicarbonate concentration < 22 mmol/l, bicarbonate supplementation had no evidence of benefit on non-kidney outcomes [72]. Nonetheless, international guidelines recommend bicarbonate repletion as a means of increasing serum bicarbonate and avoiding severe acidosis [3]. Bicarbonate repletion can be recommended to avoid a serum bicarbonate of <18 mmol/L. People with CKD and acidosis can also be encouraged to limit the intake of acid-rich foods (e.g., animal proteins) and to increase consumption of plant-based foods. 

### 4.8. Vaccinations

People with CKD are at an increased risk of infections due to their altered innate and adaptive immunity, reduced seroconversion rates after vaccination, and comorbidities including advanced age, diabetes, hypertension, cardiovascular diseases, and malnutrition [73]. The recommended vaccinations for people with CKD are outlined in Table 4. 

### 4.9. Indications to Initiate Kidney Replacement Therapy (KRT)

Despite optimal care, many people with CKD will experience progressive disease. The common indications for initiating KRT (dialysis or transplantation) are outlined in Table 5 [3]. These are relative indications that must be considered within each patient’s goals of care and symptom burden, especially since early initiation of dialysis does not confer mortality benefit [75]. In general, people with CKD may require KRT when eGFR is ~5–7 mL/min/1.73 m^2^, when hyperkalemia or hypervolemia are not able to be managed with medical therapy, or when the repletion of bicarbonate is limited by volume or the worsening of hypocalcemia [3]. A significant component of managing people with advanced CKD is preparation for KRT, as detailed in subsequent sections of this article.

### 4.10. Novel Therapies and Ongoing Investigations

While we focus here on the management of people with advanced CKD, we draw attention to ongoing investigations of novel therapies that can either delay the progression of kidney disease or can help manage several associated impairments [76]. These include agents such as proteinuria-reducing therapies, potassium-binding resins, and HIF-PHI mentioned above, as well as anti-inflammatory agents, monoclonal antibodies, and various agents which target aspects of the immune system that are involved in the pathogenesis of glomerular conditions [77]. Cost-effectiveness analyses of select agents in single care regions have been performed [78,79,80]. Additional studies are needed to consider the economic impact of each of these therapies, not only to individuals who may be limited in their abilities to pay for out-of-pocket costs of novel agents but also to healthcare systems. 

### 4.11. Use of Technologies to Support CKD Patient Care 

Digital interventions have the potential to help patients achieve recommended lifestyle modifications, improve health literacy, and promote disease self-management [81]. A smartphone support tool for physical activity and blood pressure monitoring was shown to be feasible and increased CKD knowledge [82]. A digital health intervention that offered physical activity sessions, educational videos, and peer support was also shown to be feasible and acceptable to people with CKD, though effectiveness in increasing physical activity was limited by time barriers [83]. While systematic reviews of such digital health interventions suggest that they have not had the anticipated degree of effectiveness, several additional interventions are under active investigation [84]. 

Telemedicine and remote monitoring can also assist care teams in monitoring people with CKD and can be associated with lower cost and increased accessibility for patients living in remote locations [85,86]. The increased use of telehealth after the COVID-19 pandemic has resulted in a lower reliance on specialized telemedicine equipment [87]. Furthermore, telehealth can facilitate interdisciplinary team care, which has been shown to be effective in helping CKD patients manage comorbidities such as hypertension [88].

## 5. Preparing for Kidney Replacement Therapy (KRT)

### 5.1. KRT Modalities: Dialysis, Transplant, and Conservative Care 

The incidence of kidney failure ranges from 107–213 cases per million persons per year [7], totaling 5–7 million cases of people with kidney failure requiring kidney therapy worldwide [89]. In the U.S. alone, >135,000 people are newly diagnosed with kidney failure per year [10]. Kidney replacement therapy (KRT) is a general term encompassing a range of different treatment modalities and remains the key rescue therapy for all people with kidney failure worldwide. KRT modalities include dialysis, kidney transplantation, and conservative care. Each treatment option has different advantages, limitations, and implications on survival, quality of life, and overall health status. The right choice depends on each person’s goals and values.

Dialysis: Dialysis is the most commonly used KRT modality globally. It includes hemodialysis (HD), which can be conducted in dialysis centers or at home, and peritoneal dialysis (PD), which is typically performed at home. Several clinical and social factors contribute to decisions regarding initial modality selection, including the circumstances of dialysis initiation, availability of equipment, and access to facilities that support each dialysis modality [90,91]. Dialysis modalities can significantly modify the experience and outcomes of people as they receive dialysis treatments [92]. PD is largely underutilized for a variety of reasons, including socioeconomic barriers, misconceptions about PD being an inferior form of KRT compared to HD, lack of nephrologists who are adequately trained in prescribing and managing PD, and lack of comprehensive modality education prior to the initiation of dialysis [10,93,94]. 

Transplant: Among the KRT options available, kidney transplantation is the most desired and preferred modality. It is cost-effective and provides superior clinical outcomes and better quality of life as compared to dialysis [95]. However, the scarcity of organs is a huge limitation. Most transplant candidates require some form of dialysis therapy prior to undergoing transplantation. 

Conservative care: Conservative care is the management of kidney failure without dialysis. It includes active medical management with goals of delaying the progression of kidney disease, controlling symptoms, minimizing complications, and advanced care planning [96]. It also involves anticipating the risks of decompensation and providing support for people with CKD and their care partners to maintain the best possible quality of life. Conservative management may be considered in older or frail patients who have poorer functional status and multiple comorbidities, as the survival advantage of dialysis is limited in this population [97,98]. Although data are scarce, conservative care is shown to be an appropriate treatment alternative to dialysis in select people with advanced CKD when weighing the potential benefit to quality of life against a limited anticipated survival benefit [99,100,101,102,103].

### 5.2. What Are the Benefits of Comprehensive KRT Education?

**Figure 4:**
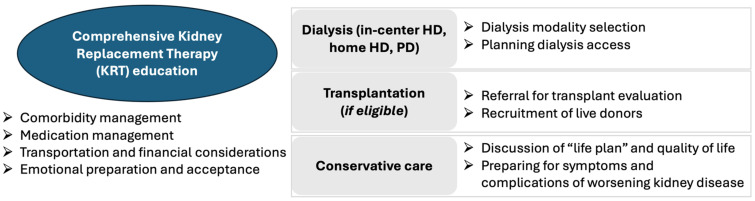
Components of comprehensive kidney replacement therapy (KRT) education. HD: hemodialysis; PD: peritoneal dialysis.

People with advanced CKD face complex medical decision-making and benefit from early education surrounding KRT modalities. Education programs can enable people with CKD to better understand kidney failure, overcome fears about dialysis, and maintain a feeling of control. People with CKD and their care teams can weigh the available treatments options and avoid emergent “crash” dialysis starts. KRT education can also allow people with CKD to process their diagnoses and share information with family members or care partners. Pre-dialysis education has been shown to reduce the incidence of urgent dialysis starts, lower mortality, and even extend the time to dialysis initiation by a median of 6 months [104,105,106,107,108,109,110]. People with CKD who receive pre-dialysis education are also more likely to choose home dialysis modalities and to receive a pre-emptive kidney transplant (prior to the initiation of dialysis) [108,111,112,113]. KRT education encompasses various components relevant to each KRT modality (Figure 4).

### 5.3. When Should People with CKD Be Referred for KRT Education?

KRT education is usually offered to people with CKD when eGFR drops < 30 mL/min/1.73 m^2^. However, people with CKD can face highly variable disease trajectories, with some people experiencing slowly progressive disease and others having a rapid decline of kidney function. Therefore, international guidelines recommend KRT planning for people who have a >3–5% risk of kidney failure over the next 5 years as calculated using a validated risk equation, a sustained fall in GFR of >20%, or a fall in GFR > 30% in the setting of initiating hemodynamically active therapies [3]. Validated risk equations can also be used to avoid unnecessary referrals for people with advanced CKD and low calculated risks of progressing to kidney failure. 

### 5.4. Role of Multidisciplinary Care Teams

Multidisciplinary care emphasizes shared responsibility for pre-dialysis education among multiple professionals. Care teams are typically composed of nephrologists, nurses, social workers, pharmacists, and nutritionists. In this model of care, each member of the team educates people with CKD in their relevant area of expertise. Team members work together, communicate with each other, and create a management plan jointly with each patient. Multidisciplinary care programs provide pre-dialysis education using a mix of one-on-one counseling sessions, group lectures, or interactive workshops. A multidisciplinary approach is an efficient and effective strategy for providing education and is associated with superior clinical outcomes after the start of dialysis therapy [114,115,116]. Implementation of multidisciplinary care is largely limited by resources available in specific care settings, and there are no standard practices for designing such clinics [117]. Some regions of Canada have established multidisciplinary clinics with risk-based thresholds for enrollment [118], whereas some practices in the U.S. explored the use of electronic consultations (e-consults) to facilitate input from multidisciplinary care team members [119].

### 5.5. Resources for Providers and People with CKD 

Kidney failure impacts people from varied backgrounds and with disparate levels of access to care. Several organizations offer educational resources, which can help support, engage, and empower people with CKD and providers with information they need to understand kidney disease, maintain health, and make informed treatment choices [120]. Additional tools can provide key information surrounding medication management, dietary support, transplantation, community resources, and more [121]. Resources for healthcare providers target different aspects of caring for people with kidney failure and provide comprehensive education to enable providers to adequately support their patients.

## 6. Equity in Transplantation: Lessons from the U.S. 

Kidney transplantation offers the optimal treatment for kidney failure with improved survival and quality of life; however, scarcity of the organ pool is a major limitation [122]. Incidence of kidney transplantation has been reported to be highest in North America, regions of Europe, and the Western Pacific [123]. The lowest rates of transplantation are found in low- and lower-middle-income countries. Some countries have more recently had a large increase in the number of living donations [124]. Unfortunately, the shortage of organ donors in many countries has been associated with unethical living donations and the trafficking of human organs [125]. There is a movement towards increasing the visibility and transparency of organ donation registries globally [126], which would also enable the evaluation of transplantation performance [127]. 

Though practices surrounding transplantation will vary across geographic regions, we call special attention to the importance of ensuring equity in transplantation. We will reference the robust data from the U.S. to explore this important topic. 

### 6.1. Current Disparities in Transplant Access

Due to this discrepancy in supply and demand, on average, 17 people in the U.S. die each day waiting for a transplant [128]. Racial and socio-economic disparities in access to organ transplantation are well-recognized [129]. Disparities exist at all steps of transplantation (including living donor kidney transplantation). These include referral for transplant evaluation, access to the national waiting list, and access to organs [130]. In a large retrospective study of over 900,000 patients who started dialysis between 2005–2014, non-Hispanic Black people had 65% lower access to transplantation compared with non-Hispanic White people in the first year after starting KRT [131]. Furthermore, Black individuals and other underrepresented minorities are less likely to be referred for transplant, including pre-emptive listing, and are less likely to complete transplant evaluation [132,133,134,135,136]. Studies have reported multiple patient, provider, and system-level factors that contribute to these disparities [137]. A systematic multi-pronged approach is needed to tackle disparities in transplant access, and a very important aspect of this effort is at the level of policymaking. 

### 6.2. Impact of the Revised Kidney Allocation System

People with CKD need to accrue time on the kidney transplant waitlist before receiving a kidney transplant. Prior to 2014, patients began accumulating time on the waitlist at the point of kidney transplant listing, which was dependent on the timing of patient referral for transplantation. Multiple studies have demonstrated racial disparities in access to the waiting list due to delayed referral and inequities in preemptive transplant waitlisting, specifically for Black and Hispanic people, women, and people with lower socioeconomic status as highlighted in the previous section. This led to lower rates of transplantation in these populations. In December 2014, a revised Kidney Allocation System (KAS) was implemented. The main aim of KAS was to decrease inequities in access to the transplant waiting list, allowing people with CKD to accrue waiting time at the start of documented kidney failure (i.e., dialysis initiation), rather than at the point of waitlisting [138]. In addition, the KAS sought greater prioritization among people who are highly sensitized (have high amounts of pre-formed antibodies against potential donors), which is another cohort that disproportionately comprises underrepresented minoritized races [139]. Short-term follow up studies of KAS have shown increased rates of transplantation in Black people and other minoritized populations [140]. In addition, the disparities in waitlisting have declined, with Black individuals having 12% lower rates of waitlisting than White individuals post-KAS, compared to 19% lower rates pre-KAS [141]. Although the total number of transplants in highly sensitized individuals increased, there was no significant improvement in racial disparities in this subset of people with CKD [142]. Additional studies are needed to evaluate the long-term impact of this change in reducing disparities. 

### 6.3. Impact of the Race-Free eGFR

In the U.S., kidney transplant waitlist eligibility is determined by eGFR. People with advanced CKD can begin to accrue wait time for transplantation after GFR decreases to less than 20 mL/min/1.73 m^2^ [143]. In most cases, GFR is estimated from equations in which race is used as a variable with the potential to overestimate kidney function in Black patients. Therefore, estimating GFR with a race coefficient may disadvantage Black people with CKD, who may face a delay in reaching an eGFR ≤ 20 mL/min/1.73 m^2^ thus making them less likely to be referred or wait-listed to receive a kidney transplant. The National Kidney Foundation (NKF) and American Society of Nephrology (ASN) established a task force in 2020 to reassess the inclusion of race in the estimation of GFR and its implications for diagnosis and subsequent management of people with, or at risk for, kidney disease [144], and the task force subsequently recommended that eGFR be calculated using a race neutral equation [13]. 

In June 2022, the Organ Procurement Transplantation Network (OPTN) adopted the recommendation of the ASN/NKF Task Force and mandated that kidney transplant programs use race-neutral eGFR calculations for all new wait list registrations [143]. This policy change, however, did not apply to Black people already on the transplant waitlist, who could have been negatively impacted by race-inclusive eGFR calculations at the time of evaluation and listing. In 2023, the OPTN Minority Affairs and Kidney Transplantation Committees subsequently enacted policy changes that created a pathway for impacted registered candidates to regain waiting time they could have received if a race-neutral calculation had been used to estimate their GFR [145]. Candidates who fulfill policy requirements can retroactively gain time with a new qualifying date of the time at which a race neutral GFR would have been ≤20 mL/min. In the first 6 months after the approval of this policy, 26% of African American waitlisted candidates gained additional wait time, of whom 8.3% received deceased donor kidney transplants [146]. Therefore, the use of race-free eGFR calculations has the potential to improve racial disparities in access to kidney transplantation [147]. 

### 6.4. Impact of Changes in National Organ Allocation Policy

In 2021, the Organ Procurement and Transplantation Network (OPTN) adopted a new allocation policy to improve organ sharing and increase equity in transplant access for all registered candidates regardless of geographic location. This policy replaced a previous policy in which the distribution of deceased donor organs was based on arbitrary donor service area and OPTN regions. According to the new policy, candidates listed at transplant hospitals within a 250 nautical mile radius (NM) of the organ procurement center will receive priority points to receive organ offers [148]. Offers that are not accepted for candidates within 250 NM will then be made for candidates beyond 250 NM. Statistical simulation modeling performed by the Scientific Registry of Transplant Recipients (SRTR) projected that the new allocation policy will improve transplant access across the country and will positively affect key groups of transplantation candidates, including children, women, ethnic minorities, and candidates who are difficult to match for immunological reasons. An early follow-up study from a single large center has shown that there was an increase in the number of organ offers and kidney transplant surgeries [149]. However, there were some concerns about the lower quality of organs, longer cold ischemia times (CIT), and increased rates of delayed graft function, all of which are potential risk factors for adverse outcomes. Additional short-term and long-term effects of this policy are undetermined at this time. 

### 6.5. Role of the Primary Care Physician

There is increasing recognition of the role of primary care physicians (PCPs) in early recognition, monitoring, basic management, and education about CKD [150]. Earlier referral to nephrology care by PCPs has been associated with increased access to transplantation and better patient outcomes [151]. Collaboration between the PCP and nephrologist, and subsequently with transplant centers, can ensure appropriate education surrounding transplantation including the referral process, work up for transplant candidacy, and outcomes. A recent quality improvement study that implemented specific tools to increase co-management of patients by the PCP and nephrologist resulted in the increased awareness of risk factors and the importance of early referral to nephrology, as well as the identification of high-risk patients [152]. Attempts to increase collaboration between PCPs, nephrologists, and transplant centers are expected to decrease disparities in transplantation given the pivotal role of the PCP in dispelling myths and mistrust about the healthcare system, which is a known factor in exacerbating these disparities.

## 7. Person-Centered Care in CKD

CKD is associated with significant morbidity and mortality, and clinicians may be inclined to focus care on the avoidance of adverse outcomes such as hospitalizations, cardiovascular events, or mortality. However, each individual with CKD experiences disease differently, and a person-centered approach may enable a greater understanding of how to guide each person with CKD through their disease process. The fundamental principles of person-centered care include attention to social, emotional, and practical needs, as well as emphasis on the utilization of shared-decision making [153]. Relevant to CKD, person-centered care can include discussions of treatment- and disease-related symptoms and advanced care planning [154]. Practices must also account for variable levels of health literacy, as low health literacy can serve as a barrier to care for patients with advanced CKD [155,156]. Interventions that tailor care to recipients can successfully improve health literacy and self-management [157,158]. 

Symptoms of CKD are substantial, with some studies citing a greater symptom burden in CKD compared to other chronic conditions including heart failure and cancer [159]. People with CKD have ranked fatigue, life participation, anxiety, and depression as key outcomes of importance [160,161], at times with higher priority than clinical outcomes such as survival [162,163]. Thus, assessment of patient-reported outcomes, which include multiple aspects of quality of life (e.g., mental, physical, and social health), is key to care of people with advanced CKD. Though symptoms can be somewhat vague and multi-factorial, evidence-based pharmacologic and non-pharmacologic strategies to manage symptoms exist and should be employed as needed [164,165,166]. Assessment of patient-reported outcomes can increase patient satisfaction [167,168] and patient activation [169], and can facilitate shared decision-making and self-management [170].

We draw attention to several qualitative and mixed methods studies that synthesize data directly from people with CKD surrounding treatment planning [171,172], advanced care planning [173], patient education for KRT planning [174], self-management in settings of limited health literacy [175], and the use of electronic health self-management tools [176]. Incorporating the perspectives of people with CKD is key to the successful implementation of management strategies. 

## 8. Summary 

While people with advanced CKD comprise a relatively small portion of the CKD population, they are faced with substantial disease morbidity and mortality and require robust multi-/inter-disciplinary support. Clinicians from multiple specialties can collaboratively manage the multifaceted comorbidities and conditions associated with CKD, including hypertension, anemia, mineral-bone disease, and acidosis. As people with advanced CKD may experience progression of their disease, collaborative care is also key in ensuring that each person is able to choose a kidney replacement therapy modality most appropriate for their needs and preferences. 

## Figures and Tables

**Table 1:** Diagnostic criteria for CKD. CKD is diagnosed in individuals found to meet any of these criteria for at least 3 months [3].

• eGFR < 60 mL/min/1.73 m^2^
• Urine albumin/creatinine ratio > 30 mg/g (≥3 mg/mmol)
• Abnormalities of urine sediment
• Hematuria
• Tubular disorders resulting in electrolyte abnormalities
• Histologic abnormality of kidney tissue
• Structural abnormality of kidney on imaging
• History of kidney transplant

**Table 2:** Suggested frequency of lab monitoring for people with CKD. CBC: complete blood count; PTHi: intact parathyroid hormone; UACR: urine albumin-creatinine ratio.

CKD Stage	Frequency of Monitoring	Labs
Low risk	Yearly	Renal function panel (including phosphorus) Urinalysis and UACR CBC (consider iron panel in people with anemia or fatigue) PTHi (consider assessment of Vitamin D levels in people with secondary hyperparathyroidism)
Moderately increased risk	Yearly	
High risk	Every 4–6 months	
Very high risk	≥ Every 3–4 months	

**Table 3:** Guideline-directed use of proteinuria-reducing therapies for adults with kidney disease [3,35,36,37,38]. * Non-steroidal MRA can be initiated for patients with eGFR ≥ 25 mL/min/1.73 m^2^. ** SGLT2i can be initiated for patients with eGFR ≥ 20 mL/min/1.73 m^2^ and continued if eGFR declines after initiation. eGFR: estimated glomerular filtration rate; GLP-1 RA: glucagon-like peptide 1 receptor agonists; MRA: mineralocorticoid receptor antagonists; RASi: renin-angiotensin-system inhibitors; SGLT2i: sodium-glucose cotransporter 2 inhibitors; T1D: type 1 diabetes; T2D: type 2 diabetes; UACR: urine albumin/creatinine ratio.

Guideline- Directed Care	Kidney Disease Improving Global Outcomes (KDIGO)	American Diabetes Association (ADA)	American College of Cardiology/American Heart Association (ACC/AHA)
RASi	People with T1D or T2D with UACR > 30–300 mg/g; Non- diabetic patients with UACR > 300 mg/g	People with T1D or T2D who have hypertension and UACR ≥ 30 mg/g	People with heart failure with reduced, preserved, or minimally reduced ejection fraction; people with chronic coronary disease
MRA	People with T2D and UACR ≥ 30 mg/g despite maximum tolerated dose of RASi *		
SGLT2i	People with T2D, heart failure, or UACR >200 mg/g **	People with T2D with cardiovascular disease	
GLP-1 RA	People with T2D who have not achieved glycemic targets despite use of metformin and SGLT2i		People with chronic coronary disease

**Table 4:** Recommended vaccinations for adults with CKD.

Vaccination	Recommendations
Hepatitis B	Vaccinate all adults with CKD G4-5 (consider vaccination in adults with CKD G1-3, especially when at high risk of disease progression) Check titers 1–2 months after vaccination series is completed, and re-vaccinate with full series if antibody titer is < 10 IU/ml
Influenza	Annual vaccination High-dose vaccines for adults ≥ 65 years
Pneumococcal	20-valent PCV (PCV20) or 15-valent PCV (PCV15), followed by 23-valent PPSV (PPSV23)
Zoster	Vaccinate adults ≥ 50 years Vaccinate adults ≥ 19 years who are planning to receive immunosuppression
COVID-19 [74]	Immunocompetent adults < 64 years old: one dose Immunocompetent adults ≥ 65 years old: two doses, at least four months apart Immunocompromised individuals: at least three mRNA vaccine doses, or at least two Novavax vaccine doses Moderately or severely immunocompromised individuals: pemivibart (Pemgarda^TM^), a monoclonal antibody authorized for COVID-19 pre-exposure prophylaxis
Respiratory syncytial virus (RSV)	Vaccinate adults > 60 years old

**Table 5:** Indications to initiate kidney replacement therapy (KRT).

Indication to Initiate Dialysis	
Symptoms attributed to kidney failure (uremic toxins)	Neurologic symptoms (confusion, lethargy) Pericarditis or serositis Anorexia or malnutrition Pruritis
Acid-base abnormalities	Acidemia
Electrolyte abnormalities	Hyperkalemia refractory to medical management
Signs or symptoms or volume excess	Volume overload refractory to diuretics Resistant hypertension

## Data Availability

Not applicable.
